# Combined Application of Fluorescence Spectroscopy and Principal Component Analysis in Characterisation of Selected Herbhoneys

**DOI:** 10.3390/molecules29040749

**Published:** 2024-02-06

**Authors:** Joanna Banaś, Marian Banaś

**Affiliations:** 1 Department of Biotechnology and General Technology of Food, Faculty of Food Technology, University of Agriculture in Kraków, Balicka 122, 30-149 Kraków, Poland; 2 Department of Power Systems and Environmental Protection Facilities, Faculty of Mechanical Engineering and Robotics, AGH University of Kraków, A. Mickiewicza 30, 30-059 Kraków, Poland; mbanas@agh.edu.pl

**Keywords:** herbhoney, spectrofluorimetry, PCA, phenolic compounds

## Abstract

This study reports the use of front-face fluorescence spectroscopy with principal component analysis (PCA) as a tool for the characterisation of selected Polish herbhoneys (raspberry, lemon balm, rose, mint, black current, instant coffee, pine, hawthorn, and nettle). Fluorimetric spectra registered in the ranges ascribed to fluorescence of amino acids, polyphenols, vitamins, and products of Maillard’s reaction enabled the comparison of herbhoney compositions. Obtained synchronous spectra combined with PCA were used to investigate potential differences between analysed samples and interactions between compounds present in them. The most substantial influence on the total variance had the intensities of polyphenols fluorescence. These intensities were the main factor differentiated by the analysed products.

## 1. Introduction

Honey and other bee products are natural food and contain natural antioxidants such as phenolic acids, flavonoids and vitamins, preventing many diseases. Their amounts and types depend primarily upon the floral source of honey. Darker honeys are usually shown to have a higher antioxidant content than lighter ones [1]. It is believed that honey has antioxidative, antibiotic, antiphlogistic and antidiabetic properties. It also positively influences red blood cells and the respiratory system. The content of phenolic compounds is the reason for the high antioxidant activity and characteristic colour [2].

Herbhoney is a honey-like substance produced by bees fed on a saccharose-based food supplemented with extracts or fruit juices. They have a wide application in pharmacy and medicine as drug components, prophylactic agents and dietary supplements [3]. Due to the use of raw plant materials of various origins, herbhoneys differ in sensory characteristics. Their chemical composition is similar to that of natural honey. The prophylactic and therapeutic action of a herbhoney is closely connected with the origin of the raw material used in its production [4]. Herbhoneys are widespread in some eastern European countries, predominantly Poland, Lithuania and Latvia. Many beekeepers in these countries produce herbhoneys for their own use and retail [5].

Traditionally, physicochemical properties have been employed for the analysis and quality assessment of honey and other bee products. Nevertheless, conventional analytical tools show limitations in a detailed analysis of product chemical composition, mechanism of occurring reactions or potential relationships between components. One of the most attractive and used methods for food analysis and quality control is near-infrared spectroscopy (NIR). It allows for fast qualitative and quantitative characterisation of products. Additionally, the continuing trend of miniaturisation of the used equipment has caused the widening of the application of this technique [6]. Fluorescence spectroscopy is the other technique with broad application in food analysis. It is also a fast method with higher sensitivity than other spectroscopic tools, with a quantification limit reaching 1 ppb; it is simple, reliable, non-destructive (similar to NIR) and economical [6,7,8]. Fluorescence spectroscopy has been validated for the quality control and analysis of a wide range of foods, including dairy products, oils and honey [9]. Fluorescence properties of honey and honey-like substances may be attributed to their phenolic constituents, amino acids, protein, vitamins, products of the Maillard reaction, and chlorophylls [7,10,11]. One of these products, hydroxymethylfurfural (HMF), is used as an indicator of honey freshness or heating [12].

Because honey and herbhoney samples contain several fluorophores, it is not sufficient to measure only a single wavelength spectrum to collect data. The synchronous fluorescence technique may also investigate such multicomponent fluorescent systems. It involves simultaneous scanning of both excitation and emission wavelengths, keeping a constant difference between them. Synchronous scanning fluorescence spectroscopy is very useful for analysing mixtures of fluorescent compounds because both excitation and emission characteristics are included into a single spectrum [13].

Fluorimetric spectra are usually too complex to be analysed or compared with naked eye. Chemometric techniques such as principal component (PCA) and linear discriminant analyses (LDA) have been used to classify wines, olive oils, juices, and different types of milk. Such techniques have also been employed in classifying honey according to its type and origin based on physico-chemical data and spectral analysis [14]. Mehretie et al. used front-face fluorimetric spectroscopy on honey samples collected from tropical regions of various floral origins for the authentication of the products. Multivariate analysis showed that honey’s fluorescence characteristics depend on their botanical origin and enabled the authenticity of the product [15].

Results of investigations on Polish herbhoneys indicate that they satisfy most of the requirements concerning genuine honey such as water, saccharose and hydroxymethylfurfural content, free acidity, and diastase number. These products also exhibit a high antioxidant activity connected with a high concentration of phenolic compounds. Due to their high phenolic compound content, they possess a high antimicrobial potential [5].

Despite the relatively good familiarity with herbhoney composition and activity, there is a need to characterise interactions between herbhoney compounds, which can influence the observed antioxidant and antimicrobial activities. The aim of this work is to investigate the use of front-face fluorescence spectroscopic data connected with PCA analysis for the characterisation of selected herbhoney samples, especially of the mentioned interactions between bioactive substances.

## 2. Results and Discussion

### 2.1. Emission Spectra in the Wavelength Range of 280–480 nm

**Figure 1:**
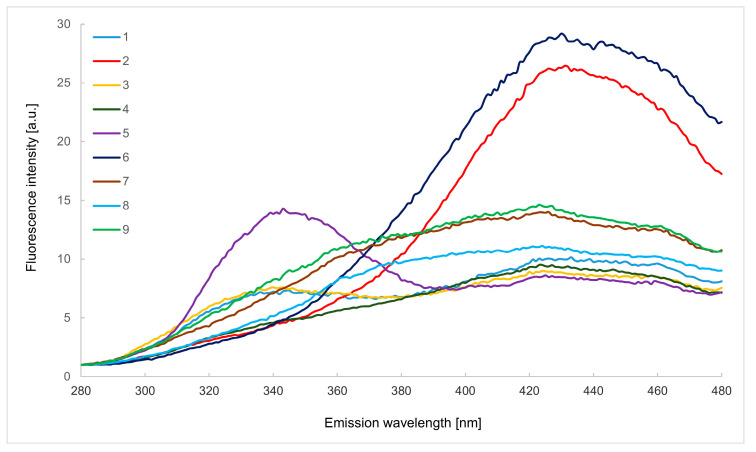
Emission spectra in the wavelength range of 280–480 nm (λ_ex_ 250 nm) of raspberry (1), lemon balm (2), rose (3), mint (4), black currant (5), coffee (6), pine (7), hawthorn (8) and nettle (9) herbhoneys.

The easiest technique to characterise samples using fluorescence spectroscopy is to record one excitation or emission spectrum. Emission spectra in the emission wavelength range of 280–480 nm (λ_ex_ 250 nm), presented in Figure 1, according to literature data, enable the observation of the fluorescence of aromatic amino acids and nucleic acids, for which the fluorescence maximum is located at approx. 340 nm [14].

Fluorescent aromatic amino acids are phenylalanine (λ = 280 nm), tyrosine (λ = 300 nm) and tryptophan (λ = 335 nm). As can be seen in Figure 1, only tryptophan exhibits fluorescence in the analysed emission wavelength range. It is known that the fluorescence of phenylalanine can be detected only in the absence of tyrosine and tryptophan, and it is also converted to tyrosine under the influence of irradiation [16]. Fluorescence bands attributed to tryptophan are visible on the spectra of black currant and, to a lesser extent, raspberry and rose herbhoneys (intensities are almost the same). The other tested herbhoneys practically do not show fluorescence bands in this range of emission wavelengths. The presence of several polyphenolic compounds is also evident, including gallic acid, vanillic acid, methyl syringate and catechin [7]. In the case of catechin (λ = 315 nm), small amounts can be found in the samples of raspberry, rose and nettle herbhoneys, while the highest fluorescence intensity was found for the sample of black currant. In this herbhoney, the highest fluorescence intensities were also recorded for the other listed phenolic compounds. Vanillic acid (λ = 330 nm) was also found in raspberry and rose samples. On the other hand, low fluorescence intensity of methyl syringate (λ = 340 nm) was noted in raspberry and rose; it was slightly higher in pine and nettle, and the highest, as already mentioned, in black currant herbhoney. The presence of gallic acid (λ = 345 nm) can be found in all analysed samples.

Other compounds whose fluorescence can be observed in the spectral range presented in Figure 1 are Maillard reaction products, such as furosine and HMF. Such compounds are bound to the proteins. According to Karoui et al., the transfer between aromatic amino acids in proteins and Maillard reaction products took place, and the bands at 360 nm can be observed [14]. The authors reported narrow peaks at 402 and 433 nm, which are related to furosine and HMF presence in the investigated samples. The main feature distinguishing herbhoney from honey is the higher content of sucrose [3], which increases the probability of forming larger amounts of HMF or furosine. These compounds are not observed in the presented works, indicating the good quality of the herbhoneys and the lack of thermal treatment before delivery to the laboratory [14].

Phenolic compound fluorescence bands are present in the emission range of 360–420 nm when excited between 250 and 280 nm [14]. Another research reported λ_max_ near 450 nm contributed by such substances as chlorogenic acid, caffeic acid and coumarins [17]. The band of caffeic acid might have been overlapping with chlorogenic and ferullic acids [18]. For this wavelength range, the highest fluorescence intensities were recorded for lemon balm and coffee herbhoney samples. The fluorescence of the other analysed products was much weaker, indicating a lower content of the compounds mentioned above.

Phenolic compounds are an important part of the antioxidant potential of honey and other bee products like herbhoneys. According to the data, the most common phenolic compounds present in honey are 4-dimethylaminobenzoic acid, caffeic acid, p-coumaric acid, gallic acid, vanillic acid, syringic acid, and chlorogenic acid [19]. Most of them are found also in herbhoneys available on the Polish market [20]. The highest total phenolic compound content (TPC) was observed in creamed honey with lemon balm (110.6 mg GAE/100 g), while the lowest was detected in hawthorn natural honey (27.27 mg GAE/100 g) [21]. The concentration of phenolic compounds in the case of herbhoneys ranged from 27.1 mg GAE/100 g (chamomile) to 75.3 mg GAE/100 g (raspberry) [20], whereas Jamróz et al. observed an amount of 116 mg GAE/100 g for lemon balm herbhoney, 134 mg GAE/100 g for instant coffee, and 110 mg GAE/100 g in case of nettle herbhoney [22]. Dżugan et al. claimed that it is result of herb additives [21].

Sinapic, gallic and chlorogenic acids indicate antimicrobial and antioxidant activity [23,24]. The dominant bioactive compounds of honey were phenolic acids, which are 87% of the sum of individual polyphenolic compounds [25]. The phenol composition of honey depends primarily on its botanical origin. The quantity of phenolic compounds can vary depending on the season of the year, climatic conditions and processing factors, as well as bee species and soil characteristics [26,27,28,29].

The results of determinations of the total polyphenol content (TPC) in the tested herbhoneys are presented in Table 1. As can be seen, the highest amount of polyphenols was found in coffee (209.38 mg/100 g) and lemon balm (126.55 mg/100 g) honey. TPCs in other products have values similar to those in the literature, in the data mentioned above, and the existing differences result from natural fluctuations in the content of bioactive ingredients in raw materials used to prepare nutrients for bees producing herbhoney.

Considering TPC values, the herbhoneys of raspberry, mint and nettle form one distinct product group, rose and hawthorn form a second one, while the other tested honeys show statistically significant differences compared to all others at *p* < 0.05. At *p* < 0.01, the difference between mint and nettle honey and rose and hawthorn herbhoneys become statistically insignificant.

### 2.2. Emission Spectra in the Wavelength Range of 305–500 nm

**Figure 2:**
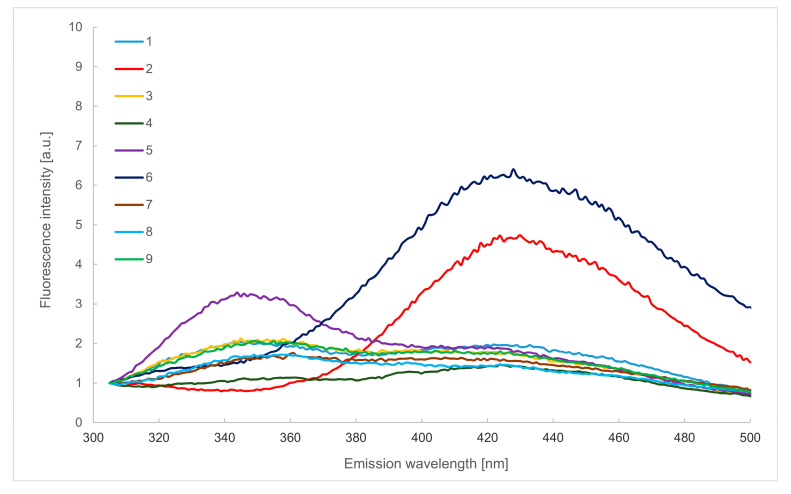
Emission spectra in the wavelength range of 305–500 nm (λ_ex_ 290 nm) of raspberry (1), lemon balm (2), rose (3), mint (4), black currant (5), coffee (6), pine (7), hawthorn (8) and nettle (9) herbhoneys.

A more detailed analysis of changes in the tryptophan content and comparison of the information obtained from Figure 1 is possible with the use of emission spectra recorded in the range of 305–500 nm after excitation at 290 nm presented in Figure 2. This spectral range enabled analysis of tryptophan residues in proteins. The highest amount of this compound was found in black currant, in mint in small amounts, and no signal was recorded in lemon balm and coffee herbhoneys.

Differences in the observed tryptophan fluorescence intensities may be caused by the type of plant used to produce the bee feed and the type of honey used. For acacia honey, the content of Trp is 33.33 mg/kg, while in honeydew honey, it is only 4.04 mg/kg [30]. In the case of lemon balm herbhoney, the lack of a band may also mean that all tryptophan is found in the internal parts of the protein structure present in this product.

According to Ali et al., low intensity of tryptophane fluorescence in stored and commercial honey samples is related to protein degradation due to long-term storage and high temperature treatment during commercial processing. This is also accompanied by a Maillard reaction to form furosine and HMF [18]. However, the presence of these compounds in the tested herbhoneys was not found by analysing the spectra presented in Figure 1.

The location of the tryptophan fluorescence band maximum offers information about the immediate surroundings of this amino acid [16]. In the case of rose, black currant and nettle samples, it was 350 nm. In contrast, for pine and hawthorn ones, the maximum value was shifted towards longer wavelengths (360 nm), which may indicate that in these products, the immediate Trp environment is more hydrophilic than in the other cases.

Significant differences in tryptophan fluorescence intensities may also be related to the content of flavonoids in these products. Among them, such compounds as quercetin, myricetin, kaempferol, luteolin, rutin, naringenin, naringin, chrysin, rhamnetin, isorhamnetin, apigenin, pinocembrin, pinobanksin, galangin, tricetin, catechin, and hesperidin are the primary compounds found in various varieties of honey [31]. It is known that some of these compounds can quench Trp fluorescence by binding to proteins, especially enzymatic ones, acting as their inhibitors [32]. Of the many existing compounds in this group, only 3-hydroxyflavone, daidzein, formononentin and ononin show native fluorescence. Flavonoids present in honey and other bee products, i.e., quercetin, miricetin, kaempferol and chrysin, are classified as substances that quench Trp fluorescence to various extents [33].

The content of flavonoids in pine herbhoney is 37.3 mg/100 g [34], while in raspberry, it is 33.58 mg/100 g and in mint it is 25.73 mg/100 g [27]. The content of individual flavonoids depends on the variety and geographical origin. Moreover, organic honey contained significantly more chrysin than the conventional variety [29]. The flavonoids of honey may originate from pollen, nectar or propolis [27]. Considering the above data, it seems that the observed differences in tryptophan intensity are related to the total content of flavonoids and the concentration of individual compounds from this group, as well as different fluorescence quenching abilities.

Moreover, herbhoney lemon balm and coffee exhibit strong fluorescence in the emission wavelength range of 420–430 nm, attributed to the B vitamins (B_2_, B_6_ and B_9_) [35]. On the other hand, the fluorescence intensity of the other tested herbhoney was relatively low, indicating a small amount of the vitamins mentioned above.

### 2.3. Emission Spectra in the Wavelength of 390–600 nm

**Figure 3:**
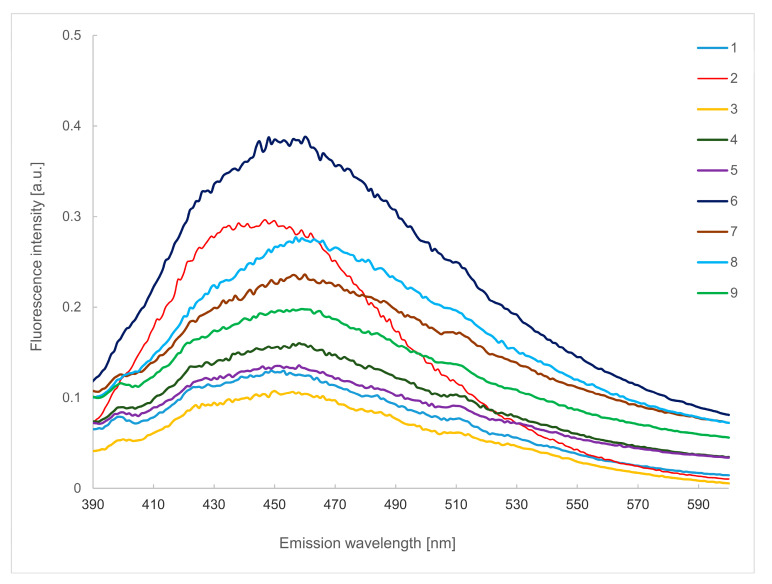
Emission spectra in the wavelength range of 390–600 nm (λ_ex_ 375 nm) of raspberry (1), lemon balm (2), rose (3), mint (4), black currant (5), coffee (6), pine (7), hawthorn (8) and nettle (9) herbhoneys.

Emission spectra recorded in the wavelength range of 380–600 nm (λ_ex_ 375 nm), presented in Figure 3, made it possible to track differences in the content of other phenolic compounds whose fluorescence could not be accurately observed in the spectral range presented in Figure 1, as well as those compounds whose fluorescence intensity was disturbed by the presence of the tryptophan fluorescence band.

The band with a maximum at 400 nm can be attributed to the presence of ellagic and ferulic acids, and its presence can be found in samples of raspberry, rose, mint, black currant, pine, hawthorn and nettle herbhoneys. The fluorescence band of caffeic acid has its maximum at 410 nm. The highest amount of it was found in lemon balm and coffee samples. In the case of chlorogenic acid, which in its pure form (without the presence of other compounds from this group) fluoresces with λ_max_ of 416 nm, a shift towards longer wavelengths (420 nm) can be seen. Its presence can be observed for all tested herbhoneys except lemon balm and coffee.

In addition, small amounts of pyridoxine (vitamin B_6_) can be detected, with a narrow fluorescence band of approximately 400 nm [35], visible in all samples except lemon balm and coffee ones. In the case of hawthorn herbhoney, this band is slightly shifted towards longer wavelengths.

A broad band with a maximum of about 450 nm can be seen in all tested samples, which can be attributed to the presence of folic acid (vitamin B_9_) [28]. In addition, in the emission wavelength range of 390–430 nm, the fluorescence band of niacin (vitamin B_3_), caffeic acid and sinapic acid [35] is also present.

According to Kunat-Budzyńska et al., caffeic and syringic acid has antibacterial and antifungal activity. In addition, caffeic acid is bactericidal against *S. aureus*. On the other hand, cinnamic acid shows antifungal properties against *A. niger*, *C. albicans* and antibacterial specimens [36]. Therefore, due to the presence of these compounds, it can be assumed that the products tested in the present work may have the listed health-promoting effects.

### 2.4. Principal Component Analysis of the Synchronous Spectra

**Figure 4:**
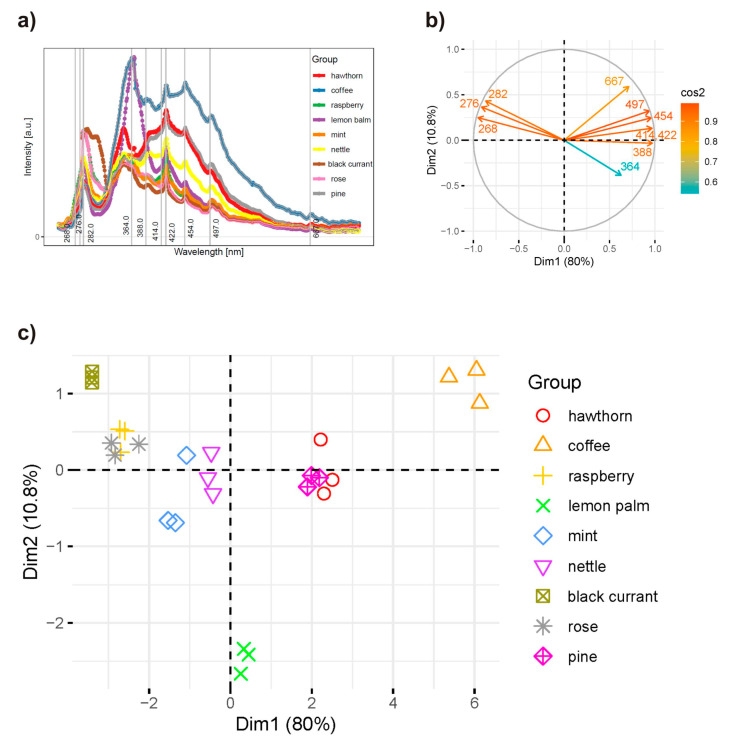
PCA results of synchronous spectra of tested herbhoneys; synchronous spectra at Δλ = 30 nm (**a**), correlation circle (**b**), score plots of PCs (**c**).

Synchronous fluorescence spectrum (SFS) is obtained by scanning both the excitation and emission monochromators simultaneously, usually with the constant wavelength interval (Δλ = λ_em_ − λ_ex_) between them. When value Δλ is chosen properly, the resulting SFS shows a few bands that are much more resolvable than those in the conventional fluorescence spectrum [37]. In the presented studies, the 10–80 nm range with a step of 10 nm was used. Synchronous spectra recorded for Δλ= 10 nm were characterised by numerous narrow bands, almost non-overlapping and with relatively high intensity. Along with the increase in the assumed wavelength difference, the broadening of the bands and a decrease in their intensity, and in many cases also in their number, were observed. Ali et al. stated that Δλ = 60 nm is optimal from the point of view of the appropriate intensity of individual fluorescence bands [18]. Principal component analysis was performed for all registered synchronous spectra. Optimal results from the point of view of interpretation of the observed relationships were obtained for Δλ = 30 nm, and they are presented in Figure 4.

Due to the incredible complexity of synchronous spectra, where the considerable overlap of fluorescence bands of individual substances is observed, it would be challenging to deduce the composition of individual samples or to identify the compounds that determine similarity or difference in a group of products of similar composition based on the spectra obtained alone. In the case of such complex spectra, they should be analysed using multivariate methods. Principal component analysis was performed on a series of synchronous spectra arranged into a matrix. It enabled the discovery of existing intrinsic structures in the data set, clustering of samples and outlier detection. This multivariate analysis was used in the present paper to obtain information on the bioactive substances, including nutritional compounds like B vitamins, in the tested herbhoneys, which significantly impact the diversity of health-promoting properties. In addition, PCA can help identify patterns, trends, and relationships among the samples that might not be apparent in the original high-dimensional data and determine which compounds enable differentiation and classification of the products among the herbhoney group. It was assumed that these phenomena are visible as differences in fluorescence intensity in the recorded synchronous spectra, and it was possible to determine the most critical factors responsible for the observed range of changes.

The three principal components, PC1, PC2 and PC3, explain 97.6% of the total variance. The first component represents an almost equal contribution of signals from all fluorescent substances in the tested herbhoneys. The second one (PC2) describes the influence of the fluorescence intensity of chlorophylls (667 nm) and, to a lesser extent, the fluorescence of phenolic compounds and tryptophan (268, 276, 282, 364 nm). In the case of the PC3 component, it mainly describes the influence of the fluorescence intensity of selected phenolic compounds (364 nm).

Figure 4b shows the strength and direction of the correlations between the fluorescence intensities of different fluorophores in the tested herbhoneys. A PCA biplot visualises the principal components derived from the original variables. The angles between vectors in a biplot indicate the degree of correlation between fluorescence intensities at particular wavelengths. Vectors pointing in the same direction suggest a positive correlation, while vectors pointing in opposite directions suggest a negative correlation. The perpendicular direction suggests a lack of correlation. By analysing the mutual position of individual vectors, it can be stated that there is a strong positive correlation between the substances for which the fluorescence maximum is located at 268, 276 and 282 nm and those at 388, 414, 422 and 454 nm. The former group includes caffeic acid, tryptophan, hydroxyl benzoic acid derivates, gallic acid, catechin and vanillic acid [38]. The second one includes fluorophores such as pigments, vitamin B_9_, and riboflavin [35].

These substances have a weaker positive relationship with the content of chlorophylls (667 nm) [13] and vitamin B_3_ (497 nm) [35], HMF as well as kaempferol and myricetin [32]. However, in the case of Maillard reaction products, i.e., furosine and HMF. Taking into account the data obtained from the analysis of the spectra presented in Figure 2, it is possible to exclude these compounds from further analysis of the impact of their content and interactions with other components on the obtained characteristics of the tested products. The group of compounds exhibiting maximum fluorescence at 364 nm is characterised by a strong negative correlation with the previously mentioned compounds fluorescing at λ_ex_ 268, 276 and 282 nm.

However, no correlation was found between the content of chlorophylls, vitamins B_2_ and B_3_ and phenolic compounds whose maximum fluorescence band is located at λ_ex_ 364 nm, as well as between the fluorescence intensity of tryptophan and polyphenols fluorescing at 268, 276 and 282 nm.

The composition of individual herbhoneys (in terms of the type and content of bioactive components) varies significantly, even at points of successive samples tested form separate groups, which is presented in the score plots of PCs (Figure 4c). The most similar compositions are observed in rose and raspberry as well as pine and hawthorn herbhoneys. Coffee, lemon balm and black currant herbhoneys show the most excellent distinctiveness in this regard. Taking into account the results of TPC determinations (Table 1), it can be concluded that in the case of samples with higher content of polyphenols, like coffee and lemon balm herbhoneys, these compounds can be used to differentiate the analysed products. The rest of the investigated samples created the groups which only partially covered the results of PCA analysis of synchronous spectra. Although TPC determination has been widely used in estimating the content of phenolic compounds in many food substances, it is not sensitive enough to accurately determine the total polyphenolic content due to interferences from other compounds present in the complex honey matrix. It indicated that in such a case, not only the content of polyphenols differentiated these samples, but also other bioactive compounds (chlorophyls, vitamins B, aromatic amino acids) played an important role. The presence of these compounds is also significant from a nutritional and health-related point of view.

Because the fluorescence intensities of the bands at 268, 276 and 282 nm and 414, 422, and 454 nm had the most significant contribution to the total variance, the content of compounds assigned to these bands had the most significant impact on the differentiation of points observed in Figure 4c. In turn, the compounds for which the fluorescence maximum is located at 364 nm had the least influence on the observed differences in the tested herbhoneys. Substances that do not seem to affect the overall characteristics of the product and the ability to distinguish individual samples are chlorophylls and vitamins B_3_ and B_9_.

## 3. Materials and Methods

The herbhoneys included raspberry (1), lemon balm (2), rose (3), mint (4), black currant (5), coffee (6), pine (7), hawthorn (8) and nettle (9) varieties, supplied by Apipol (Krakow, Poland) and Zielarz Polski (Jodłowa, Poland). Samples were not thermally treated and diluted before spectroscopic measurements.

Fluorescence spectra were registered using a Cary Eclipse spectrofluorimeter (Varian, Mulgrave, Victoria, Australia). A xenon lamp was used for excitation. Excitation and emission slit widths were 5 nm. Measurements were performed using a quartz cuvette with 1 cm optical length in a front-face mode using a Solid Sample Holder (Varian, Mulgrave, Victoria, Australia). The incidence angle of the excitation radiation was set at 56°.

Using excitation wavelengths of 250, 290 and 375 nm, the fluorescence emission spectra were recorded in the wavelength ranges of 280–480 nm, 305–500 nm and 390–600 nm, respectively [14].

Synchronous fluorescence spectra were collected by simultaneously scanning the excitation and emission in the range of 240–750 nm with the constant Δλ between them. The spectra were recorded for Δλ in the 10–80 nm range every10 nm. All of the spectra were registered in triplicate with change in cuvette orientation at each measurement.

The total content of polyphenols was determined by the method of Singleton et al. using the Folin–Ciocalteau reagent [39] in 10% water extracts of the herbhoneys. Absorbance was measured in a spectrophotometer (Cecil CE 9500, Cecil Instruments, Cambridge, UK) at a wavelength of λ = 750 nm, and the results were expressed as mg of gallic acid per 100 g of product. All measurements were performed in triplicate.

Differences between the mean values were determined using Tukey’s test. Differences were considered statistically significant at *p* < 0.05 and *p* < 0.01. Comparisons were made using CSS Statistica v. 12.5 (Stat Soft Inc., Tulsa, OK, USA).

Numerical analyses of fluorescence spectra were carried out using original procedures written in the R language (version 4.0.2), using FactoMineR library packages [40] for PCA analysis and FactoExtra [41] for graphical presentation of the obtained results. The recorded synchronous fluorescence spectra showed the dependence of fluorescence intensity I on excitation wavelength λ and the type of herbhoney sample tested. Due to the noise of the output spectra, they were smoothed using the Savitzky–Golay algorithm [42]. The next stage of the analysis was to reduce the amount of data by selecting a set of fluorescence intensities at the excitation wavelengths corresponding to the maxima of individual bands, which was achieved based on a differentiated spectrum analysis. The sets of input data for PCA analyses were matrices with dimensions z × p, where z is the number of variables and p is the number of cases.

## 4. Conclusions

Spectrofluorimetry is a research method that allows very accurate characterisation of herbhoneys. Its application made it possible to analyse the differences in the content of most polyphenols in the tested products, especially when the synchronous spectra were considered. Using PCA analysis for the synchronous spectra enabled us to determine the compounds whose fluorescence band is present on the registered spectra and the extent to which individual sample differentiation and characterisation is allowed. Knowing the qualitative composition of polyphenols, especially caffeic acid, hydroxyl benzoic acid derivates, gallic acid, catechin and vanillic acid, and their total content in the tested herbhoneys, it was possible to conclude their pro-health properties. Based on PCA results, the relationships between individual polyphenols, pigments, and vitamins B_3_ and B_9_ could be found. By comparing the fluorescence intensity of tryptophan and the location of the band maximum, it was possible to determine the immediate environment of this amino acid in the tested products and indirectly make a conclusion about the presence of flavonoids that quench its fluorescence, such as quercetin, myricetin, kaempferol and chrysin.

## Figures and Tables

**Table 1:** Results of the total polyphenol content (TPC) of the tested herbhoneys.

No	Sample	TPC (mg GAE/100 g)
1	raspberry	54.49 ± 1.10 ^a,A^
2	lemon balm	126.55 ± 1.21 ^b,B^
3	rose	64.77 ± 0.32 ^c,C^
4	mint	49.52 ± 0.96 ^a,A,C^
5	black currant	32.86 ± 1.78 ^d,D^
6	coffee	209.38 ± 4.04 ^e,E^
7	pine	86.62 ± 0.93 ^f,F^
8	hawthorn	64.21 ± 5.11 ^c,C^
9	nettle	57.11 ± 1.42 ^a,A,C^

Values in a column denoted with different letters are significantly different (*p* < 0.05, small letters; *p* < 0.01, capital letters).

## Data Availability

Data are contained within the article.
